# A Smile

**Published:** 1880-10

**Authors:** J. J. Keyes


### A SMILE.

J. J. KEYES.

I would like to describe a smile anatomically, so to speak; to state all of its physiological conditions. But I can not do it. Were I not a hundred and twenty miles from the "Institute" I suspect I could get the information requisite for such a description from the doctor with the black hair, the wise head, and the smiling eyes. Webster defines a smile as a "peculiar contraction of the features of the face which naturally expresses pleasure, moderate joy, approbation, or kindness." He also says it is an expression of countenance resembling a smile, but "indicative of opposite feelings." Henry Ward Beecher has this definition: "It is the color which love wears, and cheerfulness and joy—these three: it is the light in the window of the face, by which the heart signifies to father, husband, or friend that it is at home and waiting."

Both definitions are sentimental, the latter a little more poetically expressed than the former. Indeed, you cannot describe a smile prosaically; something poetic will creep into your definition. If I say a smile is a contraction of certain facial muscles, I have, to be sure, made a scientific, un-poetic definition, accurate so far as it goes; but it is incomplete; and to make it complete I must add something sentimental, because a certain class of sentiments must exist in the mind and be pictured on the face, to produce a genuine smile. Webster calls them "pleasure, moderate joy, approbation, kindness." Mr. Beecher grows poetic and calls them "cheerfulness," "the color which love wears," "the light in the window of the face." A sunbeam is capable of rigid, scientific analysis and definition; and yet a sunbeam is full of poetry full of sentiment, and affords more real satisfaction to the mind thus considered, than when examined with the spectroscope and made the subject of chemical analysis.

A smile is a ray from the soul, lighting up the features of one's face; and I am not sure that I care for an accurate, scientific definition of it. Even though I could have it, I should prefer to regard it as the soul's ray, and look at it from the sentimental side. There is, to me, something so poetic, so beautiful and so expressive in a smile, that all I wish or care to know about it is what feeling of the heart, what motion of the soul, what thought of the mind, it pictures on the countenance. It is a phenomenon that never appears except on the human face. A dog may give signs of gladness; so may a cat, or a cow, or a horse; but no animal ever wore an expression corresponding closely to the smile of the human face. From this I infer that men and women either have a higher, finer class of emotions, or else have a pleasanter method of betraying them; for there is nothing, not even language, capable of such wide and varied ranges of expression as the human countenance; and its smiles—ah! they light the way to some of the deepest and innermost recesses of the soul!

But, first, let us glance for a moment at the *stereotyped* smile. Now and then we meet a person who seems to be always smiling. A smile seems to be stamped upon his face, as certain figures are stamped upon various fabrics. When we meet him for the first time, we have an impression that he must be an extremely pleasant person; but when we become acquainted, we are disappointed to find that the smile is only on the face, and not at all in the soul; and we are reminded of that horrible caricature of Victor Hugo's, "The Man Who Laughs." The smile signifies anything or nothing, chiefly nothing. I knew such a man once, in a certain church. I at first supposed that he was an agreeable, good natured man, who liked everybody, and was pleased with everything. But the smile on that man's face was stereotyped. He was born with it; and there it was, even when he was the most solemn, or when he was plotting the most outrageously against his friends and against the peace of the church to which he belonged. One soon learns to distinguish a smile like that, and to despise it. At all events, one ceases to regard it as significant of any pleasurable or other sentiments in the soul of him who wears it.

Let us notice briefly some varieties of the genuine smile. We will begin with the lowest and least attractive. First, there is the *cynical* smile. A cynic is said to be a follower of Diogenes, who was a snarler, a growler, a man who was pleased with nothing. The word cynic is from a Greek word meaning dog; but the real cynic owes an apology to the dog; for the dog is sometimes good natured, pleased with his condition and surroundings, and does his best to give us a real, beautiful, dog-smile. But of course he sometimes snarls and barks.

Did my readers never see the cynic smile his smile of derision and contempt? Did you never see the man-dog snuff, and snarl, and growl, with his nose elevated, and a satanic grin spread over his features?—a smile that conveyed naught but the smiler's contempt for you, or your weakness; for society, for culture, for beauty, for riches or pleasures. I imagine Diogenes must have worn such a smile when he walked through the streets of Athens with a lighted candle, saying that he was in search of a "man." And when he had finished the search he, no doubt, still smiled and growled, and growled and smiled and said: "There, now! I told you so. There is not a man in Athens." The cynical smile is not attractive.

Neither is the *sinister* smile. The sinister smile bodes no good. It shadows forth an evil design, or a cruel purpose. It indexes the baser, darker passions of the human soul. It rests upon the face of the villain who plots the ruin of unsuspecting innocence. It lights up the face of him who plans revenge against his enemy. The sinister smile, if it is meant for you, expresses the purpose of doing you some injury, or of "paying you off" for some insult, or damage, or offence. The sinister smile is sardonic, black, cruel, and essentially hellish; and mirrors forth a class of sentiments that no noble soul will for a moment entertain.

In the stereotyped, cynical and sinister smile we see nothing pleasing. The first offends us by its insincere and meaningless character; and the other two by the unworthy state of mind and the revolting sentiments which they indicate. Let us notice still others. We often see the smile of *incredulity*. Sometimes it is pleasing, and sometimes offensive to us. But you never fail to detect it. It has as marked and definite a character as the cynical smile. You are making a statement which seems somewhat large to your friend. Unconsciously to yourself, you exaggerate, overstate, or color too highly some object, or fact, or description. Glancing at your friend's face, you see that he is receiving your story with many grains of allowance. He has not said a word, perhaps. He has neither turned away his head nor made any deprecating motion with his hand, nor said, "Oh, dear; now!" He has only smiled. But in that smile you saw doubt; you saw that his mind was questioning the accuracy of your statements. And you were offended.

You are a man of large plans and visionary ideas. And you have a quick-witted wife who knows you pretty well. And you are telling her your plans. You explain to her how the money can be piled up in heaps; how Pactolian streams can be made to run all around your dooryard; how your little wife will then never have to soil and harden her hands with work any more. And that little wife smiles upon you. She knows you are talking nonsense; and by the way she smiles, and then exchanges glances with the children, you are made aware that she regards your plans and expectations as the airiest kind of air castles. It is the incredulous smile, and a very significant smile it is. It needs not the aid of language. Its meaning is unmistakeable. The mind thus displays its wonderful power. It imparts to the features an expression which says as plainly as words could say it: "I do not believe you"; or "I must regard your statements as doubtful."

But we now come to something better and more attractive than anything we have considered,—the smile of genuine *good nature*. Ah, there are, thank God, some, yea many, genial, sunny souls in the world. Happy souls, trusting souls they are, who see something beautiful in the world, and good in human nature. And they do not go through life with clouded face and wrinkled brow, displeased with everybody and everything, shocked at people's short-comings, criticising people's motives, and bemoaning day and night the sins of their neighbors. There are too many such men and women in the world. And when we meet, as we sometimes do, people whose natures are unsoured, who are filled and running over with good cheer and good fellowship, who are conscious, every day, of the joy of living, and whose happy, shining souls beam beautifully in their smiling faces, we are in love with them right away. We cannot help ourselves. We do not wish to help ourselves. We find in the smile of genuine good nature a light which dissipates our gloom, an inspiration which gives to us new faith and courage for the work and struggle of life, and a source of such gladness and contentment as make us feel that life is not wholly a sorrow nor a cheat. Sunny souls are the light of the world, and their smiles, unforced and free, are exhilarating and comforting to other souls.

Once more ; we have seen the smile of *love.* How can it be described ? How can we put into words the unspeakable ! The smile of love is divine and inexpressible. It far surpasses the smile of good nature. It plays only upon the face of him or her whose soul has been touched with a celestial fire. I do not speak of love in any gushing, sentimental sense, as set forth in silly romances. A true, high born love is not inane and senseless. Its conditions I cannot stop to mention ; but its character is divine. Love is longing ; love is confidence ; love is sympathy ; love is uniting ; love is joy, and sometimes pain ; love loves, and sometimes adores. And the smile of love may have in it all the *elements* of love. We have seen the mother gazing on her sleeping babe, while a smile of ineffable tenderness and sweetness overspread her face. It was the smile of love. We have seen that same mother, years later, bid her boy a smiling good bye, when she knew he was going to the drinking saloon, the gaming table, or to the place of evil resort. It was the smile of love ; but the love was longing, and the longing was pain ; and the smile ought to have broken the boy's heart.

We have seen the soul speaking still another language with its smile of love. It was when the features of some true man and noble woman were all radiant with smiles that spoke of confidence, perfect faith, perfect repose, a mutual gladness, a mutual satisfaction, a union to remain unbroken forever !

Between man and man, also, when a manly love exists, we have seen the smile of love exchanged, and felt its pleasant thrill. David and Jonathan were like lovers, and no doubt often looked smilingly into each other's faces—saying not a word. Oh ! this smile of love ! How beautiful, attractive, and soul-soothing it is ! How thrilling, sometimes, to catch the glance and smile of one who believes in you and loves you ! And how hapless the lot of those whose souls have never known this joy, or having known it, now know it no more ! Alas for them !

The subject is not exhausted ; it is only begun. But it can be pursued no farther in this article. I conclude, and my readers will conclude that the soul is a wonderful thing, and has a wonderful power. There is scarcely a feeling, passion, or sentiment which it cannot express on the human face ; and a larger number of these than I have time to enumerate, may be expressed by that simple thing,—a smile. Let the smile be as often as possible the smile of genuine good nature and the smile of love. Let us fill up with sunshine, and then radiate for the comfort and happiness of others. Let us beam and glow with the "color that love wears, and cheerfulness and joy,—these three."

